# Body and Soul; or Life, Mind, and Matter Considered as to Their Peculiar Nature and Combined Condition in Living Things, &c.

**Published:** 1847-04-01

**Authors:** 


BODY AND SOUL; OR, LIFE, MIND, AND MATTER, CONSIDERED AS TO THEIR PECULIAR NATURE AND COMBINED CONDITION IN LIVING THINGS, &c. By *George Redford,* M.R.C.S., &c. Octavo, pp. 232. London, Churchill, 1847.

SUCH as our readers as are partial to metaphysico-physiological enquiries will be pleased, we should think, with this work upon the whole. A variety of questions connected with the manifestations of Vital and Mental Action are successively brought forward and discussed. Among these, Phrenology attracts a good deal of notice; its claims are examined [but not with perfect fairness], and declared to be for the most part visionary. Here is a specimen of our author's reasoning:—

"The general fact of the brain being the organ of the mind is admitted by all; and we may perhaps venture to say, that the hemispheres are the especial organs of the intellect. Yet if we attempt to go beyond this, we verge upon hypothesis: however, phrenologists boldly tell us, that the anterior portions of the hemispheres are the seat of intellect, and the superior of the 'sentiments,' which, by the way, is a rather obscure word, while the posterior are devoted to the propensities or animal faculties. We will forego the discussion of the psychology of such an arrangement, to point out that this would be anatomically wrong, for the posterior or animal regions are deficient in some lower animals; a sheep, *e. g.*, possesses no posterior lobes of its brain, only the middle and anterior, therefore it should be only intellectual and sentimental; a sufficient absurdity. The posterior part of the brain in man is commonly larger than the anterior; and in comparison with the same parts of the brain in lower animals, is much larger in proportion than the same part in any of them; but we do not find that man at all equals the lower animals in the care and '*love of offspring*' or '*inhabitiveness,*' the love of particular places, all of which faculties are located by phrenologists in the posterior parts of the brain; again, in children the most prominent parts of the brain are those to which the faculties of '*reasoning*' and '*caution*' are allotted, which is rather paradoxical; the proper explanation of the fact being, that those parts of the brain appear exceedingly prominent on account of the large proportionate size of the brain and the smallness of the face, the greatest length and breadth of the brain being at those points." P. 189.

